# Mechanisms of FA-Phagy, a New Form of Selective Autophagy/Organellophagy

**DOI:** 10.3389/fcell.2021.799123

**Published:** 2021-12-07

**Authors:** Jiayi Lu, Bernard Linares, Zhen Xu, Yan-Ning Rui

**Affiliations:** Department of Neurosurgery, McGovern Medical School, The University of Texas Health Science Center at Houston, Houston, TX, United States

**Keywords:** focal adhesion, autophagy, cargo receptor, organellophagy, cancer, vascular integrity, intracranial aneurysm

## Abstract

Focal adhesions (FAs) are adhesive organelles that attach cells to the extracellular matrix and can mediate various biological functions in response to different environmental cues. Reduced FAs are often associated with enhanced cell migration and cancer metastasis. In addition, because FAs are essential for preserving vascular integrity, the loss of FAs leads to hemorrhages and is frequently observed in many vascular diseases such as intracranial aneurysms. For these reasons, FAs are an attractive therapeutic target for treating cancer or vascular diseases, two leading causes of death world-wide. FAs are controlled by both their formation and turnover. In comparison to the large body of literature detailing FA formation, the mechanisms of FA turnover are poorly understood. Recently, autophagy has emerged as a major mechanism to degrade FAs and stabilizing FAs by inhibiting autophagy has a beneficial effect on breast cancer metastasis, suggesting autophagy-mediated FA turnover is a promising drug target. Intriguingly, autophagy-mediated FA turnover is a selective process and the cargo receptors for recognizing FAs in this process are context-dependent, which ensures the degradation of specific cargo. This paper mainly reviews the cargo recognition mechanisms of FA-phagy (selective autophagy-mediated FA turnover) and its disease relevance. We seek to outline some new points of understanding that will facilitate further study of FA-phagy and precise therapeutic strategies for related diseases associated with aberrant FA functions.

## Introduction

A focal adhesion (FA) is a large macromolecular assembly consisting of the transmembrane protein integrin connected to the actin cytoskeleton through adaptor proteins and intracellular signaling molecules (Burridge, 2017). Integrins in FAs bind to different ligands, such as collagen or fibronectin, in the extracellular matrix (ECM), and thus mediate the attachment of cells to their ECM. As subcellular compartments that localize at the ventral membrane of the cells, FAs can sense environmental cues, such as ECM rigidity, as fewer FAs have been observed on softer matrices (Yeh et al., 2017). Although FAs were originally observed in two-dimensional culture dishes, they exist in different cell types *in vivo* and are critical adhesive organelles for mediating cell-ECM interactions (Lo and Chen, 1994; Geiger et al., 2001; Delon and Brown, 2009; Kuo et al., 2012).

In the cerebrovascular system, FAs play an essential role in preserving vascular stability by adhering the monolayer endothelium to its basement membrane that is predominantly composed of ECM proteins such as collagens and laminins. Inactivation of the αv or *β*8 integrin subunit of FAs in mice (McCarty et al., 2002; Proctor et al., 2005) or the integrin-binding partner talin in zebrafish (Wu et al., 2015), leads to cerebral hemorrhage. Interestingly, our previous study showed that thrombospondin type 1 domain containing 1 (THSD1), a single-span transmembrane protein, physically interacts with talin and integrin (Rui et al., 2017). Loss of THSD1 also causes cerebral hemorrhage in both zebrafish and mice as well as contributes to intracranial aneurysm development in humans (Santiago-Sim et al., 2016), highlighting the role of FAs in cerebrovascular diseases.

In addition to their role in vascular stability, FAs are broadly linked to the initiation and progression of various cancers. Focal adhesion kinase (FAK) as a central protein of FAs promotes FA turnover and cell migration and stabilization of FAs by FAK inhibitors was shown to have anti-tumor effects (Schober et al., 2007; Tiede et al., 2018). As dynamic organelles, FAs are tightly controlled by different signaling pathways and can be asymmetrically distributed inside cells. For instance, FA turnover rate is significantly higher in the leading edge than the rear area of the migrating cells and inhibition of FA turnover blocks cell migration and prevents the cancer dissemination in mice models (Sharifi et al., 2016; Chang et al., 2017), suggesting that FAs may serve as a promising therapeutic target for cancer metastasis, a devastating stage of this disease that accounts for over 90% of cancer patient deaths.

FA is controlled by its formation and turnover. In comparison to numerous publications on FA formation, few studies elucidate the mechanisms that regulate FA turnover. Recently, autophagy, a self-eating system in the cells, was reported to degrade FAs. In this paper, we mainly review the mechanisms of autophagy-mediated FA turnover, a subject that has recently attracted intensive attention. Knowledge from this emerging research field may provide novel therapeutic targets for cerebrovascular diseases or more broadly for cancer that is associated with the loss of FAs.

## Selective Autophagy of FA Turnover/FA-phagy

Autophagy is a catabolic process that degrades intracellular cargo in the lysosome. There are three types of autophagy: macroautophagy, microautophagy, and chaperone-mediated autophagy (Yu et al., 2018). In comparison with the latter two, macroautophagy is a process in which double-membrane vesicles termed autophagosomes form and sequester substrates. Our review focuses on macroautophagy because macroautophagy (hereafter referred to as “autophagy” for simplicity) has the capacity to degrade large cargo. In autophagy, autophagosomes marked with LC3 protein on both sides of the membrane can engulf the large cargo, such as mitochondria or protein aggregates, and subsequently fuse with the lysosomes for cargo degradation (Lamark and Johansen, 2012; Palikaras et al., 2018).

Depending on the mechanisms of cargo recognition by the autophagosomes, autophagy can be categorized as selective and non-selective. Selective autophagy depends on a specific organelle receptor while non-selective does not. Starvation induces non-selective or bulk autophagy. The main purpose of this biological process is to survive adverse conditions by recycling necessary cellular constituents and generating nutrient support such as amino acids. Many autophagy-specific genes such as *atg5* and *atg7* that are essential for assembling autophagosomes, the core machinery in autophagy, were identified from genetic screens in budding yeast under starvation conditions (Yu et al., 2018). In addition to surviving unfavorable conditions, autophagy plays a fundamental role in preserving cellular hemostasis that is important for health. Autophagy can degrade damaged organelles like endoplasmic reticulum (ER) or mitochondria or excessively large cargo, such as protein aggregates or nuclear pore complexes (NPC) (Lamark and Johansen, 2012; Lee et al., 2020; Ma et al., 2020; Yang et al., 2021). Notably, autophagy-mediated turnover of ER, mitochondria, protein aggregates or NPC is a selective process, where cargo receptors are required to bring the autophagosomal membrane to the cargo sites for engulfment and degradation. For instance, different cargo receptors were identified for selective autophagy-mediated ER turnover, suggesting that distinct mechanisms may exist for controlling ER homeostasis under different stresses (Stolz and Grumati, 2019; Yang et al., 2021). In some scenarios, a large scaffold protein such as huntingtin is involved and facilitates selective autophagy, a process we outlined in a previous publication (Rui et al., 2015). Depending on the substrates, selective autophagy is referred to as NPC-phagy, ER-phagy, aggrephagy, mitophagy, and lipophagy, respectively (Table 1).

**TABLE 1:** Types of selective autophagy/organellophagy and cargo receptors.

Types of selective autophagy	Selective cargos	Cargo receptors
NPC-phagy	Nuclear Pore Complex (NPC)	Nup159
ER-phagy	Endoplasmic Reticulum (ER)	FAM134B,SEC62,RTN3,CCPG1,TEX264,ATL3,PGRMC1,ATG39,ATG40
Aggrephagy	Aggregates	P62,NBR1
Mitophagy	Mitochondria	NIX,BNIP3,FUNDC1,SPATA33,Atg32
Lipophagy	Lipid Droplet	P62
FA-phagy	Focal Adhesion (FA)	NBR1,c-Cb1

Autophagy was recently reported to degrade FAs in different cell types. First, autophagosomal marker LC3 was found to co-localize with the FA protein paxillin in human breast epithelial cells or mouse embryonic fibroblasts (Sharifi et al., 2016; Kawano et al., 2017). The co-localization rate is higher in the leading edge of the migrating cells where FAs are more frequently degraded. Second, knockdown of autophagy essential genes such as *ATG5* or *ATG7* increases both the number and the size of FAs, supporting the idea that autophagy negatively regulates FA stability (Kenific et al., 2016; Sharifi et al., 2016). Importantly, autophagy-mediated FA turnover is a selective process and two cargo receptors including NBR1 and c-Cbl were identified (Kenific and Debnath, 2016; Chang et al., 2017), respectively. Another study demonstrated that the cargo recognition step can be regulated by SRC kinase, though the identity of the cargo receptor is unknown (Sharifi et al., 2016). Based on all aforementioned published results, we coined the term “FA-phagy” for selective autophagy of focal adhesions (bottom line in Table 1), according to its analogous role with other types of selective autophagy such as ER-phagy or NPC-phagy.

## Cargo Recognition Mechanisms in FA-Phagy

Selective autophagy has two main steps: autophagosome biogenesis and cargo recognition. The latter step has become a more attractive therapeutic target than the former in autophagy cascade. Autophagic core machinery that generates autophagosomes is shared in all types of selective autophagy, while the cargo recognition mechanism can be distinct and specific. So far, more than a dozen cargo receptors have been identified for different forms of selective autophagy (Table 1). Therefore, targeting cargo recognition mechanisms in disease should be more precise than general inhibition of autophagosomes biogenesis that may affect other types of selective autophagy essential for the quality control of respective organelles, which would have undesired consequences.

**FIGURE 1:**
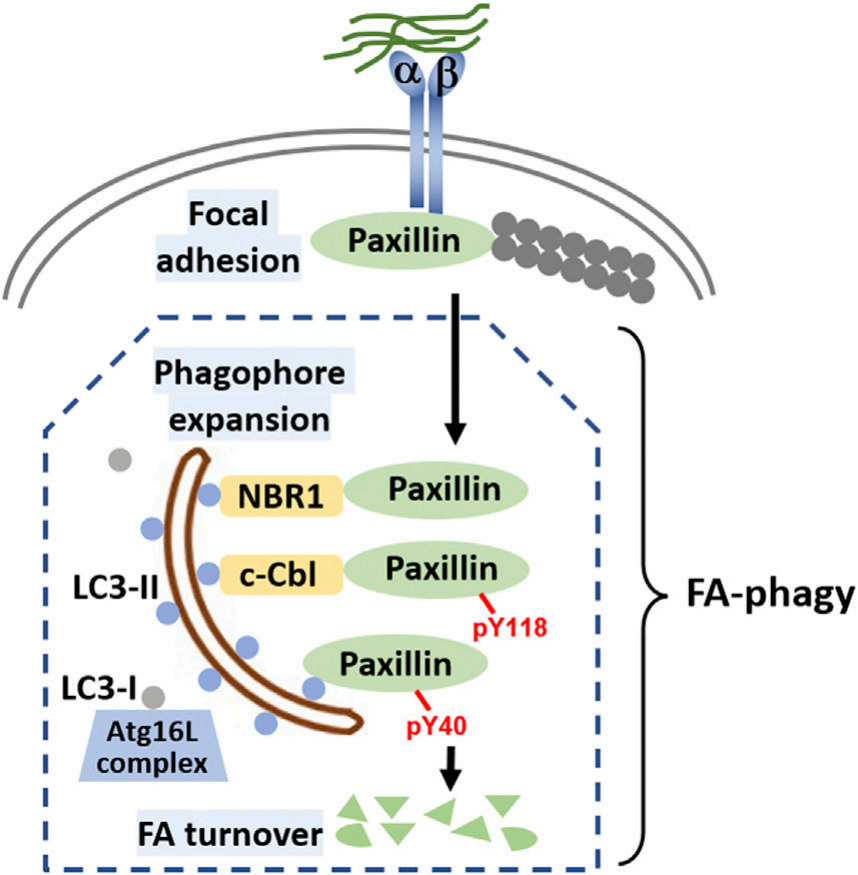
Molecular mechanisms of FA-phagy. Focal adhesions (FAs) are simplified as protein complexes containing integrin *a* and *ß* that bind to both the extracellular matrix and intracellular paxillin protein. Filamentous actin (F-actin) comprising actin monomers are in grey and are attached to paxillin. Different cargo receptors such as NBR1 or c-Cbl, on one hand directly interact with LC3 (round blue dots associated on both sides of autophagosome), and on the other, recognize cargos such as paxillin, facilitating FA-phagy. Alternatively, paxillin can be phosphorylated at tyrosine 40 in an SRC-selective manner, which increases the binding affinity to LC3. In the cells, LC3 mainly has two forms, LC3-I and LC3-II. With the help of Atg16L complex, LC3-I is transformed to LC3-II by a lipidation process. Lipidated LC3-II is able to insert into both sides of the phagophore membrane, a well-established mechanism for autophagosome around the cargo. LC3-II on the inner side of the autophagosome will be degraded by lysosomal enzymes after enclosed autophagosome fuse with lysosome for cargo degradation.

In FA-phagy, NBR1 is the first cargo receptor that was identified by Debnath’s group (Kenific et al., 2016). NBR1 is an adaptor protein with multiple domains, including a LC3-interacting region (LIR). As a cargo receptor, NBR1 links autophagosomal membrane to FAs by directly interacting with both LC3 and paxillin, two prominent markers for each organelle, respectively. Notably, the NBR1 mutant lacking LIR lost the ability to induce FA-phagy. Later, Alaoui-Jamali’s team demonstrated that c-Cbl, an E3 ligase with a ring-finger domain, functions as another cargo receptor in FA-phagy (Chang et al., 2017). A new LC3-interacting region was mapped out in c-Cbl that is known to bind paxillin. Interestingly, c-Cbl preferentially recognizes Y118-phosporylated paxillin, compared to Y31-phosphorylated paxillin, which adds the posttranslational modification as a layer of regulation in cargo recognition step. In addition, Macleod’s lab reported that SRC as a non-receptor tyrosine kinase regulates the cargo recognition efficiency in FA-phagy (Sharifi et al., 2016). In particular, SRC phosphorylates paxillin at Y40, which creates a new LC3 binding motif. Y40-phosphorylated paxillin can be engulfed by autophagosomes by interacting with LC3. Consistently, paxillin mutant Y40F that is resistant to SRC-mediated phosphorylation becomes more stable in FA-phagy. Cargo recognition mechanisms in FA-phagy are altogether cell type-specific (summarized in Figure 1), possibly due to the differential expression level of cargo receptors in different cells or the posttranslational modification codes on substrates.

## FA-Phagy as a Therapeutic Target

So far, two reports have demonstrated that inhibiting FA-phagy prevents cell migration and cancer metastasis. Increased number of FAs was observed in breast cancer cells when the core autophagy machinery was inactivated by knockdown of *ATG5* or *ATG7*, which retards the metastasis to lung and liver (Sharifi et al., 2016). Another study showed that FA turnover in cancer cells was blunted by interfering with c-Cbl-mediated cargo recognition efficiency and thus cell migration and progression to metastasis was inhibited (Chang et al., 2017). These data support the idea that FA-phagy can serve as a target for treating cancer metastasis.

Virtually no studies have reported on the role of FA-phagy in cerebrovascular diseases in comparison with its known contribution to cancer progression. Interestingly, autophagy was upregulated in the samples of patients with intracranial aneurysms and loss of FAs and cerebrovascular integrity has been observed during intracranial aneurysm development (Lee et al., 2015; Wang et al., 2015; Santiago-Sim et al., 2016; Rui et al., 2017). It would be intriguing to explore the hypothesis that FA-phagy negatively regulates vascular integrity and examine whether cargo receptors like NBR1 or c-Cbl play an essential role in cerebrovascular diseases, such as intracranial aneurysm or many other diseases due to the loss of vascular integrity.

## Conclusion/Discussion

Altogether, FA-phagy is a selective process even though cargo recognition mechanisms can be distinct and context-dependent, allowing for an exquisite regulation in FA stability. Posttranslational modifications such as tyrosine phosphorylation may play an important role in the cargo recognition step and cargo with different modifications may have differential binding affinity to cargo receptors. This idea was supported by the observation that c-Cbl preferentially recognizes Y118-phosphorylated paxillin but not Y31-phsphorylated paxillin in FA-phagy (Chang et al., 2017). It is worth mentioning that both c-Cbl and NBR1 have a ubiquitin-associated domain (UBA) that interacts with ubiquitin, a well-documented posttranslational modifying molecule that can label the substrates for degradation via proteasome system or autophagy. NBR1 was reported to bind ubiquitinated protein aggregates by its UBA domain and is required for aggrephagy (Lamark and Johansen, 2012). Some FA proteins can be ubiquitinated such as integrin, paxillin, and VASP (Iioka et al., 2007; Lobert and Stenmark, 2010; Boyer et al., 2020). Therefore, ubiquitination may serve as another mechanism for regulating cargo recognition specificity and/or efficiency in FA-phagy; however, more experiments are needed to test this possibility.

In FA-phagy, as in other selective autophagy types, cargo receptors both recognize the cargo to be degraded, potentially by binding to the substrates or to the added moieties in the substrates caused by posttranslational modification, and recruit the autophagosomal membrane to the cargo. One of the prominent features for cargo receptors is that they can physically interact with LC3 connecting autophagosomal membranes to the cargo. However, the origin of this autophagosomal membrane remains a mystery. Are they formed locally or transported to the FAs from elsewhere? It is possible that common vesicle trafficking routes are involved since autophagosomes were reported to transport via the microtubule network by kinesin/dynein motor proteins or via actin filament by myosin proteins (Kimura et al., 2008; Tumbarello et al., 2012). It is equally possible that autophagosomes can form *de novo* at the cargo site, a phenomenon that has been observed in a few recent investigations. Phagophores that lack LC3 can act as a membrane resource for autophagosomal formation. Such phagophores can exist in some organelles to be degraded, such as p62-positive condensates or damaged mitochondria. Notably, FIP200 localizes at the phagophores and interacts with both autophagy initiation kinase ULK1 and cargo receptors such as p62 or NDP52 in aggrephagy or mitophagy, respectively (Turco et al., 2019; Shi et al., 2020). Once LC3 replaces FIP200 at the phagophores, autophagosomes begin to mature, expand, and eventually engulf the cargo. These results suggest an exciting new model in which on-demand autophagosome biogenesis can be initiated by cargo in selective autophagy. Intriguingly, FIP200 was originally identified as FAK-interacting protein by a yeast-two hybrid screen and confirmed as a functional regulator for FAK in FA stability (Abbi et al., 2002). It would be tempting to examine whether FIP200 plays a role in FA-phagy especially at the cargo recognition step.

In comparison to other types of selective autophagy/organellophagy, FA-phagy is poorly understood with many unknown facets remaining to explore. FAs generally comprise two types: small, dot-like nascent ones and large, streak-like mature ones. Are the mature FAs preferentially degraded by autophagy while the nascent ones are alternatively degraded by the proteasome system? Furthermore, are there any other cargo in FA-phagy besides paxillin? Since FA is a large macromolecular assembly, it is likely that other structural components can be recognized by cargo receptors. Zyxin might be an interesting candidate since it mainly labels the mature FAs, which are much bigger than the nascent ones and has a higher demand on autophagy-mediated turnover. These new FA components may require different cargo receptors that can physically bind to them as well as to autophagosomal protein LC3 for FA disassembly or digestion. Furthermore, in addition to phosphorylation and ubiquitination, there might exist other types of posttranslational modifications, such as acetylation or sumoylation, which fine-tune the cargo recognition step in FA-phagy in response to different stimuli. Wound-healing triggers FA-phagy in cancer cells but more other upstream signals are still awaiting to be identified. Last but not least, how does dysregulated FA-phagy contribute to human diseases such as intracranial aneurysm development or cancer metastasis? More mechanistic studies of FA-phagy in cell culture and animal models may reveal exciting new insights that may establish FA-phagy as a therapeutic target for these devastating diseases.
